# Improving sensitivity, specificity, and reproducibility of individual brainstem activation

**DOI:** 10.1007/s00429-019-01936-3

**Published:** 2019-08-21

**Authors:** Eva Matt, Florian Ph. S. Fischmeister, Ahmad Amini, Simon D. Robinson, Alexandra Weber, Thomas Foki, Elke R. Gizewski, Roland Beisteiner

**Affiliations:** 1 Department of Neurology, Medical University of Vienna, Spitalgasse 23, 1090 Vienna, Austria; 2 High Field Magnetic Resonance Centre, Medical University of Vienna, Spitalgasse 23, 1090 Vienna, Austria; 3 Institute of Psychology, University of Graz, Universitätsplatz 3, 8010 Graz, Austria; 4 Department of Biomedical Imaging and Image-guided Therapy, Medical University of Vienna, Spitalgasse 23, 1090 Vienna, Austria; 5 Department of Neuroradiology, Medical University of Innsbruck, Anichstraße 35, 6020 Innsbruck, Austria

**Keywords:** Brainstem, Trigeminal motor nucleus, Reproducibility, Reliability, Physiological noise, Functional magnetic resonance imaging

## Abstract

Functional imaging of the brainstem may open new avenues for clinical diagnostics. However, for reliable assessments of brainstem activation, further efforts improving signal quality are needed. Six healthy subjects performed four repeated functional magnetic resonance imaging (fMRI) sessions on different days with jaw clenching as a motor task to elicit activation in the trigeminal motor nucleus. Functional images were acquired with a 7 T MR scanner using an optimized multiband EPI sequence. Activation measures in the trigeminal nucleus and a control region were assessed using different physiological noise correction methods (aCompCor and RETROICOR-based approaches with variable numbers of regressors) combined with cerebrospinal fluid or brainstem masking. Receiver-operating characteristic analyses accounting for sensitivity and specificity, activation overlap analyses to estimate the reproducibility between sessions, and intraclass correlation analyses (ICC) for testing reliability between subjects and sessions were used to systematically compare the physiological noise correction approaches. Masking the brainstem led to increased activation in the target ROI and resulted in higher values for the area under the curve (AUC) as a combined measure for sensitivity and specificity. With the highest values for AUC, activation overlap, and ICC, the most favorable physiological noise correction method was to control for the cerebrospinal fluid time series (aCompCor with one regressor). Brainstem motor nuclei activation can be reliably identified using high-field fMRI with optimized acquisition and processing strategies—even on single-subject level. Applying specific physiological noise correction methods improves reproducibility and reliability of brainstem activation encouraging future clinical applications.

## Introduction

The brainstem is a complex anatomical structure which is densely packed with functionally specialized nuclei involved in the propagation of sensory and motor signals, pain modulation, and autonomic processes. The brainstem plays a critical role in various disorders including autonomic dysfunctions (Brook and Julius 2000), affective disorders (Paul and Lowry 2013), chronic pain conditions such as migraine (Denuelle and Fabre 2013), and movement disorders such as Parkinson’s disease (Braak et al. 2003; Holiga et al. 2015; Tison and Meissner 2014). However, due to its anatomical peculiarities, investigations of brainstem functions with neuroimaging techniques are challenging and further efforts for improving signal quality are needed to ensure valid and reliable assessments of brainstem activation. The aims of the present study were to investigate the feasibility of ultra-high-field functional magnetic resonance imaging (fMRI) to detect brainstem activation in single subjects as needed for clinical diagnostics and to evaluate various physiological noise correction schemes.

Most of the functional imaging studies targeting the human brainstem have investigated pain perception and modulation (Cahill and Stroman 2011; Dunckley et al. 2005; Fairhurst et al. 2007; Ghazni et al. 2010; Hahn et al. 2013; Khan and Stroman 2015; Nash et al. 2009; Ritter et al. 2013; Schulte et al. 2016; Youssef et al. 2016). Given the importance of the brainstem in the propagation of sensory information, several studies have been conducted addressing auditory (De Martino et al. 2013; Hawley et al. 2005), tactile (Ghazni et al. 2010; Nash et al. 2009; Zhang et al. 2006), and visual perception (Limbrick-Oldfield et al. 2012) including oculomotor control (Linzenbold et al. 2011; Ruehl et al. 2017). Further research targets encompass the vestibular system (Wildenberg et al. 2011), respiratory control (Faull et al. 2015; Pattinson et al. 2009), emotion processing (Satpute et al. 2013), reward prediction (D’Ardenne et al. 2008), and consciousness (Gili et al. 2013). Motor tasks such as finger tapping and facial muscle contractions have been used as functional localizers (Faull et al. 2015), to systematically test the impact of different preprocessing methods (Beissner et al. 2011), or to evaluate the impact of physiological noise correction (Harvey et al. 2008). Resting-state approaches have identified brainstem networks and their associations with cortical regions, resulting in descriptions of the vestibular circuitry (Kirsch et al. 2016), motor and autonomic networks (Bianciardi et al. 2016), the connectivity of the dopaminergic system (Vytlacil et al. 2014), as well as a potential pontine portion of the default mode network (Beissner et al. 2014).

Physiological noise in fMRI is generally taken to refer to changes in the MR signal caused by the subject’s physiology, excluding the neuronal activation of interest, in particular signals related to cardiac and respiratory cycles (Brooks et al. 2013; Jezzard 1999). Cardiac activity induces periodic changes in the cerebral blood flow and volume (Krüger and Glover 2001), as well as arterial pulsation that directly affects the BOLD signal in surrounding tissue (Piché et al. 2009) and induces oscillatory movements in the cerebrospinal fluid (Friese et al. 2004). The respiratory cycle has been shown to cause changes in the static magnetic (*B*_0_) field (Raj et al. 2001) and in the arterial CO_2_ partial pressure (Wise et al. 2004). Due to its anatomical location—surrounded by the cerebrospinal fluid (CSF) and in direct vicinity of large arteries—the brainstem is particularly affected by physiological noise (Beissner 2015; Brooks et al. 2013; Dagli et al. 1999; Harvey et al. 2008).

One commonly used method to reduce physiological confounds is to record cardiac and respiratory signals and to model these as low-order Fourier series (RETROICOR; Glover et al. 2000) that can be either used to correct EPI time series in the course of preprocessing (Faull et al. 2015; Gili et al. 2013) or to generate nuisance regressors in the general linear model (GLM). However, there have been several suggestions about the optimal number of regressors derived from physiological recordings (Harvey et al. 2008; Hutton et al. 2011; Verstynen and Deshpande 2011) mirrored by a range of methods to include physiological information in statistical models (Beissner et al. 2014; Cahill and Stroman 2011; Limbrick-Oldfield et al. 2012; Pattinson et al. 2009; Schulte et al. 2016; Wildenberg et al. 2011). Another approach is to extract physiological signal variations directly from functional images, such as Independent Component Analysis (ICA) or anatomical Component-Based Noise Correction Method (aCompCor, Behzadi et al. 2007). ICA decomposes the signal into components with similar patterns and is able to identify both large-scale brain networks and components related to physiological noise or scanner artifacts (Beckmann and Smith 2004; Beissner et al. 2014). However, the selection of appropriate components is difficult and lacks objectivity in case of manual definition or is prone to misclassifications in automated classification methods (Brooks et al. 2013). That might explain why ICA has, so far, only been applied in a limited number of studies targeting the human brainstem (Moher Alsady et al. 2016; Beissner et al. 2014; Holiga et al. 2015). In contrast to ICA, aCompCor uses a priori assumptions of the anatomical source of physiological noise signals. As it is unlikely that signal variations in the CSF or the white matter derive from neuronal activity—but rather from subject motion, respiration, and cardiac functions—these can be regressed out of the BOLD signal in statistical models. While this method is commonly used in cortical functional connectivity analyses (Whitfield-Gabrieli and Nieto-Castanon 2012), only few studies have applied aCompCor or comparable methods in brainstem fMRI (Hahn et al. 2013; Kirsch et al. 2016; Vytlacil et al. 2014).

Another possibility to use anatomical information to reduce the effect of physiological fluctuations on the BOLD signals of interest is to exclude areas prone to such fluctuations from the analysis. By cropping functional images to the brainstem (Beissner et al. 2014; Nash et al. 2009; Youssef et al. 2016) or to even smaller regions such as the periaqueductal gray (Satpute et al. 2013) before smoothing, one can avoid the smearing of unwanted signal (stemming in particular from the CSF) into regions of interest. Beissner et al. (2014) demonstrated that applying a brainstem mask before conducting ICA with resting-state data resulted in noticeably better detection of brainstem nuclei activation and reduced artifacts.

Rather than including physiological parameters in the statistical models, an appealing approach is to minimize their influence during image acquisition. Several MR acquisition strategies have been proposed to minimize the influence of physiological noise. These include head motion restriction (Edward et al. 2000), cardiac gating (Beissner et al. 2011; D’Ardenne et al. 2008; Hawley et al. 2005; Zhang et al. 2006), field monitoring (https://onlinelibrary.wiley.com/doi/epdf/10.1002/mrm.25303), and multi-echo EPI acquisition (Kundu et al. 2012; Beissner et al. 2011). Given that the brainstem nuclei constitute very small structures of interest—on the scale of some millimeters—many studies have recommended the use of high spatial resolution (Beissner 2015; Brooks et al. 2013). Reduced voxel size leads to lower signal-to-noise ratio (SNR), so this needs to be compensated for by either increasing the number of volumes, applying parallel imaging techniques, or using higher field strength (Beissner 2015). Parallel imaging techniques such as multiband facilitate the increase of the temporal resolution that is important for an adequate sampling of cardiac fluctuations, which have a typical frequency of about 1 Hz and respiratory signals that oscillate at approximately 0.3 Hz (Brooks et al. 2013). Hahn et al. (2013) demonstrated in a direct comparison between 3 T and 7 T fMRI that the superiority of higher field strength is particularly evident in brainstem regions such the periaqueductal gray. However, some drawbacks of high-field imaging need to be considered. First, susceptibility-related field inhomogeneities increase with field strength, leading to geometric distortions of EPI images (Sladky et al. 2013) that need to be corrected, for example, using either static *B*_0_ field mapping and distortion correction (Jezzard and Balaban 1995; Robinson and Jovicich 2011) or dynamic distortion correction (Dymerska et al. 2018). Second, physiological noise, including subject motion and cardio-respiratory functions increases with the square of the static field strength, while the SNR only increases approximately linearly (Krüger and Glover 2001; Triantafyllou et al. 2005). Increasing the spatial resolution and applying moderate smoothing partly accounts for this issue by increasing the temporal SNR at 7 T in particular when including motion and physiological noise regressors in the statistical model (Hutton et al. 2011; Triantafyllou et al. 2006).

Following these suggestions, we applied an EPI sequence optimized for brainstem imaging with high spatial and temporal resolution achieved using multiband acquisition at ultra-high field (7 T).* B*_0_ field inhomogeneities were corrected using* B*_0_ distortion correction via field mapping (Jezzard and Balaban 1995; Robinson and Jovicich 2011). Furthermore, all statistical models included subject motion as nuisance regressors, as motion has been shown to be the dominant source of noise at 7 T (Hutton et al. 2011). As there are several suggestions for reducing physiological noise, we compared the most common and promising techniques comprising RETROICOR and aCompCor-based approaches in combination with automatized CSF and manual brainstem masking procedures. We systematically investigated the impact of the differential physiological noise correction methods on measures of sensitivity and specificity of brainstem nuclei activation. In light of the current debate concerning the reproducibility of fMRI results (Eklund et al. 2016; Nichols et al. 2017; Slotnick 2017), we repeated the measurements four times for every subject to estimate the impact of different physiological noise correction methods on the reproducibility and reliability of brainstem fMRI.

## Materials and methods

### Data acquisition

Six healthy participants (3 female, mean age 29.7) participated in 4 fMRI sessions with a median interval of 13 days between measurements (range 6–58 days). Jaw clenching was used as motor task that has previously been shown to elicit activation in the trigeminal motor nucleus (Beissner et al. 2011) offering a clear a priori hypothesis of the expected anatomical location of task-related activation. Subjects were instructed to repeatedly clench their teeth (with a frequency of about 1 Hz) without opening the mouth, thereby producing only minimal task-related motion. In each session, the participants performed 8 runs of the task, with each run consisting of three motor blocks and four rest periods of 20 s. The study was approved by the local ethics committee and all participants gave their written informed consents according to the Declaration of Helsinki.

### Measurement parameters

The measurements were performed with a 7 T MAGNETOM system (Siemens Healthcare, Erlangen, Germany) using a 32 channel Nova Medical head coil (Wilmington, USA). 140 volumes were acquired per run using an optimized multiband EPI sequence with 30 coronal slices parallel to the floor of the IVth ventricle (TR/TE = 1000/23 ms, multiband factor = 2, in-plane acceleration = GRAPPA 2, echo spacing = 0.81 ms, bandwidth = 1450 Hz/Px, FOV 220 × 220 mm, 1.46 × 1.46 mm in-plane resolution, 1.5 mm slice thickness, 15% gap, with the phase-encoding direction being foot-to-head). *B*_0_ field maps were acquired before the functional scans with the same slice prescription as the EPI sequence using a multi-echo gradient-echo (MGE) sequence (TR 800 ms, TE 5, 10, 16 ms). Cardiac and respiratory waveforms were recorded using Siemens Bluetooth Physiological Monitors. For the pulse oximetry (PO), a photoplethysmograph with an infra-red emitter was placed on the left index finger. The respiratory sensor was attached to a pneumatic belt wrapped around the abdomen and was placed medially beneath the diaphragm. PO and respiration were both recorded at a sampling frequency of 50 Hz and were time-locked to the first volume acquired in each run.

### Image preprocessing

**Fig. 1:**
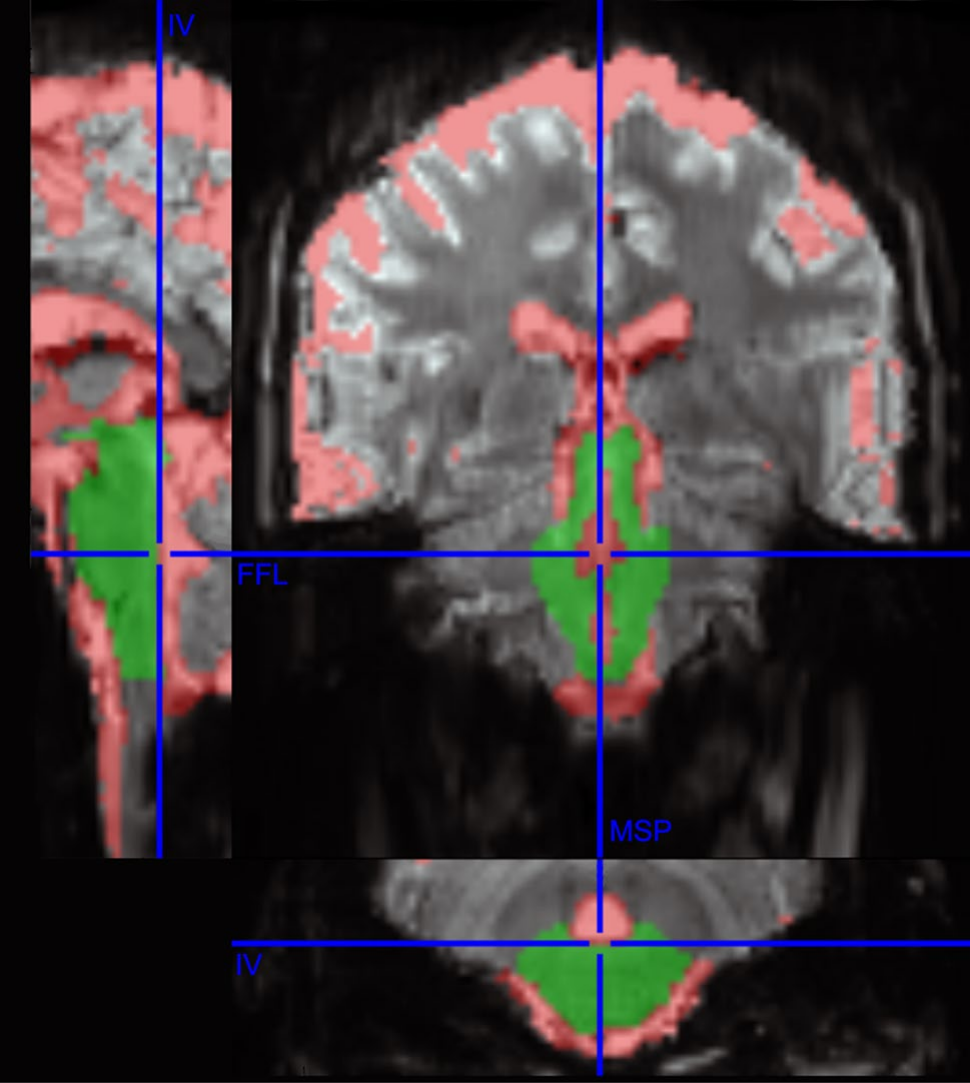
Individual cerebrospinal fluid (CSF, red) and brainstem (green) masks overlaid on the mean EPI (Subject 2) with the stereotactic axes mid-sagittal plane (MSP), the IVth ventricular floor plane (IV), and the fastigial floor line (FFL). The automatically created CSF mask was used for extracting aCompCor nuisance regressors and as an exclusive mask for the CSF masked analysis (data set ii). The manually delineation of the brainstem was used to create an inclusive mask for data set iii

Data preprocessing was performed with SPM12 (Wellcome Trust Centre for Neuroimaging; www.fil.ion.ucl.ac.uk/spm/software/spm12). B_0_ field maps were generated from multi-channel multi-echo phase data which were unwrapped using UMPIRE (Robinson et al. 2014). No slice timing correction was applied and it has been shown to have no benefit for a block design in combination with a TR of 1 s (Sladky et al. 2011). For each subject, all runs of one session were realigned to the mean volume of the session. The resulting motion parameters were used to generate nuisance regressors for the first-level analysis and to compute the voxel displacement (VD) as the root-mean-square of the translation parameters (VD = sqrt(x^2^ + y^2^ + z^2^), Van Dijk et al. 2012). The field map acquired in the respective session was coregistered to the session-specific mean volume and then used for distortion correction of the realigned functional data (Robinson and Jovicich 2011). Subsequently, the realigned and distortion-corrected images of each session were coregistered for each subject separately. Thus, all functional images including the mean EPI over all sessions were in the same subject-specific space. Spatial normalization was omitted to avoid registration errors which may have biased the results. Data were smoothed using a 3 mm FWHM Gaussian kernel to increase the temporal SNR (Triantafyllou et al. 2006). As smoothing leads to a superimposition of confounding signals from the CSF on adjacent brainstem signals, smoothing was performed with different masking procedures: (i) without masking; (ii) with prior masking of CSF regions; and (iii) with prior masking of the brainstem and images cropped to the masked regions (Fig. 1). The individual probabilistic CSF mask derived from SPM segmentation of the coregistered mean EPI (thresholded at 0.25) and was applied to the coregistered functional images by excluding all voxels within the CSF mask. The individual brainstem mask comprising medulla, pons, and mesencephalon was delineated on the individual coregistered mean EPI using MRIcron and then applied as an inclusive mask to the functional data.

### Statistical analysis

First-level statistical analyses were performed with SPM12 and included a high-pass filter with the SPM default value of 128 s. Data sets i–iii comprising the three different masking procedures were analyzed using a general linear model (GLM) with the activation blocks convolved with the hemodynamic response function as regressor of interest, six motion parameters derived from the realignment as movement nuisance regressors (Friston et al. 1996) and different physiological nuisance regressors. Specifically, physiological data were either extracted from the CSF mask using aCompCor (Behzadi et al. 2007) or directly calculated from physiological recordings using the PhLEM toolbox Version 2.0 (http://sites.google.com/site/phlemtoolbox/; Verstynen and Deshpande 2011). Three sets of regressors were extracted from the pulse and respiration recordings (**R**):

- R3: Basic regressors from the physiological recordings
  The first set of regressors were generated by applying PhLEM default settings (Verstynen and Deshpande 2011), including one regressor from the pulse recordings (PO) extracted by filtering (0.002–0.12 Hz; Butterworth filter) and down-sampling the raw signal, and two respiration regressors derived from the respiration recordings. The respiration regressors comprised the sine and the cosine value for the primary respiratory frequency (first order Fourier term).
- R8: RETROICOR regressors
  Corresponding to the Glover et al. paper introducing RETROICOR (2000), the first- and second-order Fourier terms for both physiological noise sources (PO and respiration) were extracted with the PhLEM toolbox, resulting in 8 noise regressors (the sine and cosine values for each order of each source).
- R14: Extended RETROICOR regressors
  Following the suggestions of Hutton et al. (2011) for ultra-high-field fMRI, sine and cosine Fourier series components extending to the third harmonics for respiratory and cardiac signals (6 terms for each signal source) were combined with variations in breathing volume (RV; Birn et al. 2006) and heart rate (HR; Chang et al. 2009). These calculations were performed using the default settings of the PhLEM-toolbox for phase signals, RV and HR parameters (Verstynen and Deshpande 2011) including convolution of the RV signal with the respiration response function (RV + RRF, Birn et al. 2008) and the HR signal with the cardiac response function (HR + CRF, Chang et al. 2009) modelling the effect of HR and RV on the BOLD signal.

Corresponding to the TR of the EPI sequence (1000 ms), all regressors were down-sampled to 1 Hz to generate appropriate regressors of no interest for the first-level statistical analysis.

Similar to the physiological recording approach, three sets of regressors were extracted from the CSF using the aCompCor (Behzadi et al. 2007) implementation in the REX toolbox (http://web.mit.edu/swg/software.htm).

- A1: CSF time series
  For the first aCompCor-based method, time-series data in the CSF mask were extracted using the ‘scaling’ option of the REX toolbox, resulting in one nuisance regressor. The time courses of all voxels within the CSF mask were averaged and scaled within runs to percent signal change referenced to the mean value of the CSF mask.
- A3 and A6: CSF eigenvariates
  For the other aCompCor-based methods, the eigenvariates of the signals within the CSF mask were extracted with the REX toolbox, once with three dimensions (A3) and once with six dimensions (A6).

In summary, physiological noise correction methods comprised three aCompCor strategies (A1, A3, and A6) and three regressor sets derived from physiological recordings (R3, R8, and R14). A first-level model without any physiological nuisance correction was also calculated, resulting in seven physiological noise correction approaches. Overall, 21 first-level analyses (combinations of the three masking procedures with the seven nuisance regression methods) were calculated for each session of each subject including the “base model” (no masking, no physiological noise correction). The resulting SPM T-maps for the contrast of interest (jaw clenching vs. rest) were subjected to a region of interest (ROI) analysis followed by an estimation of sensitivity and specificity using a receiver-operating characteristic (ROC) analysis.

### ROI and ROC analyses

For each subject, individual bilateral anatomical regions of interest (ROI) of the trigeminal motor nucleus were delineated on the subject-specific mean EPI using MRIcron by EM and inspected by RB. The stereotactic coordinates published by Beissner et al. (2011) were used to define the exact anatomical location of the trigeminal motor nucleus, describing its position relative to three anatomical axes (the mid-sagittal plane, the IVth ventricular floor plane, and the fastigial floor line) that were well identifiable on the mean EPI (Fig. 1). A bilateral area in the high pons (HP) containing no motor or sensory nuclei according to the Duvernoy’s Atlas of the Human Brain Stem and Cerebellum (Naidich et al. 2009) was selected as a control ROI. Both target and control ROI comprised 3 × 3 × 4 voxels per side (approximately 4.5 × 4.5 × 6 mm) resulting in bilateral ROIs of 72 voxels (~ 240 mm^3^).

Individual ROIs were transferred to the individual SPM T-maps of each session. Positive *T* values and the percentage of activated voxels (FDR 0.05 corr.) within the target and control ROIs were calculated for all subjects and sessions and all masking and physiological noise correction methods. Voxelwise *T* values entered the ROC analysis (SPSS 24) with the label of true positive in case of a positive *T* value within the target ROI and the label false positive in case of a positive *T* value in the control ROI. The area under the curve (AUC) value is the primary outcome of the ROC analysis, as it represents the combination of sensitivity (true positives) and specificity (1—false positives).

To test if the target and control regions are per se (without masking or nuisance regression) comparable regarding the extent to which they were affected by physiological noise and signal distortions, temporal SNR (tSNR) was calculated for the realigned, distortion corrected, and coregistered functional data. tSNR has been shown to capture temporal noise features including cardiac and respiratory cycles, making it appropriate to quantify fMRI signal quality (Parrish et al. 2000). First, voxelwise tSNR was calculated as the mean signal divided by the standard deviation of the signal over the detrended time series, where detrending was performed by subtracting a second-order polynomial fit to the global mean signal (Robinson et al. 2008). Second, tSNR values were extracted from individual target and control ROIs and averaged over subjects and sessions.

### Reproducibility and reliability analysis

Reproducibility was assessed by calculating the overlap of activated voxels between the sessions for each subject individually. The overlap ratio (OR) quantifies the reproducibility of the activation location, originally by comparing the activation in two measurements (Rombouts et al. 1997):

$${\text{OR}}\, = \, 2\times \left( {{\text{A}}_{\text{overlap}} } \right)/\left( {{\text{A1}}\, + \,{\text{A2}}} \right).$$

A_overlap_ represents the number of voxels that are active in both sessions and A1 and A2 the number of voxels that are activated in the first (A1) and in the second measurement (A2). The OR was adapted for calculating the Overlap Index (OI) for four sessions:

$${\text{OI}}\, = \,[ 2\times \left( {{\text{A}}_{{ 2 {\text{Sessions}}}} } \right)\, + \, 3\times \left( {{\text{A}}_{{ 3 {\text{Sessions}}}} } \right)\, + \, 4 {\text{ x }}\left( {{\text{A}}_{{ 4 {\text{Sessions}}}} } \right)]/\left( {{\text{A1}}\, + \,{\text{A2}}\, + \,{\text{A3}}\, + \,{\text{A4}}} \right).$$

A_xSessions_ is the number of voxels that is activated in exactly × sessions, for example, in only two sessions for A_2Sessions_. Similar to the original OR, the OI ranges between 0 representing no overlap and 1 for a perfect overlap between the sessions. The OI was calculated for the target and the control ROI using an activation threshold of FDR 0.05 corrected.

To assess the impact of the different processing methods on the reliability between subjects and sessions, an intraclass correlation (ICC; Shrout and Fleiss 1979) was conducted with SPSS 24. Two activation measures, the percentage of activated voxels (FDR 0.05 corr.) and the mean *T* values in the target nuclei, entered a two-way mixed ICC(3,1) for absolute agreement.

## Results

### ROI analysis

**Fig. 2:**
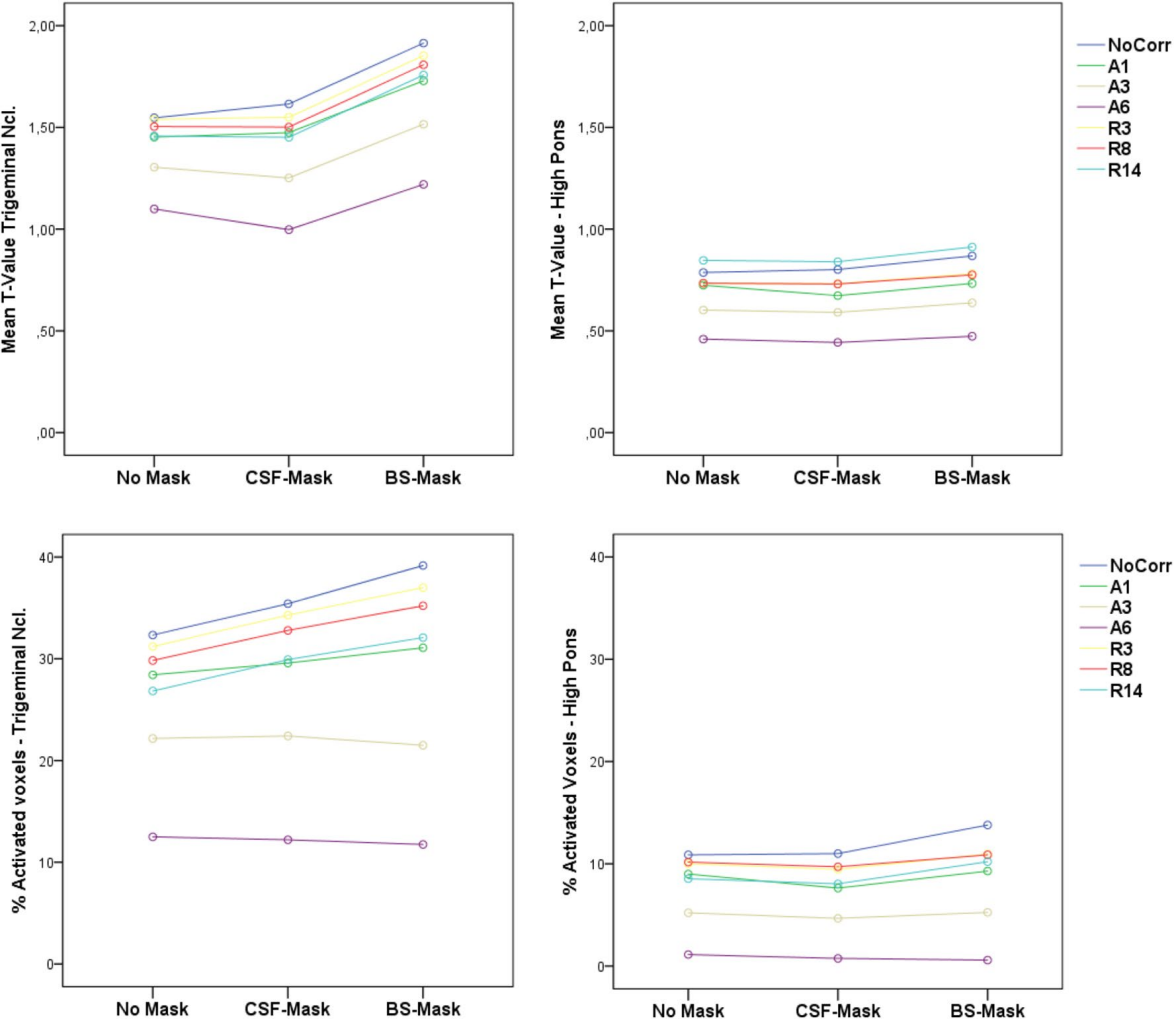
Mean *T* values and percentage of activated voxels (FDR 0.05) for the target (Trigeminal Ncl.) and control ROI (High Pons) for all masking and physiological noise correction methods across subjects and sessions. *CSF*  Cerebrospinal fluid, *BS* brainstem, *A* aCompCor, *R* RETROICOR

The average activation measures (mean *T* values and the percentage of activated voxels over all subjects and sessions) for the target (trigeminal nucleus) and control ROI (high pons) are depicted in Fig. 2. For both variables of interest, a clear difference between target and control ROI was observed across all masking and correction methods, with higher values for the target ROI. The masking procedures increased the sensitivity to trigeminal activation (in particular the brainstem mask), whereas the mean *T* values and the percentage of activated voxels in the high pons were relatively unaffected by the masking. The physiological noise correction methods generally led to decreased activation that was comparable between the target and control ROI. While including physiological recording-based regressors (R) and aCompCor with 1 regressor (A1) in the GLM model resulted in a reasonable sensitivity for trigeminal activation, the aCompCor eigenvariates (A3 and A6) as nuisance regressors led to a very low sensitivity (lower than 23% of activated voxels for A3 and lower than 13% for A6). tSNR was comparable in the trigeminal (mean = 31.93, SD = 3.26) and the control ROI (mean = 30.33, SD = 2.57) over all subjects and sessions. As these calculations were based on the distortion corrected and coregistered data without any masking or nuisance regression a biasing influence of general signal differences between the target and control ROIs can largely be ruled out.

### ROC analysis

**Fig. 3:**
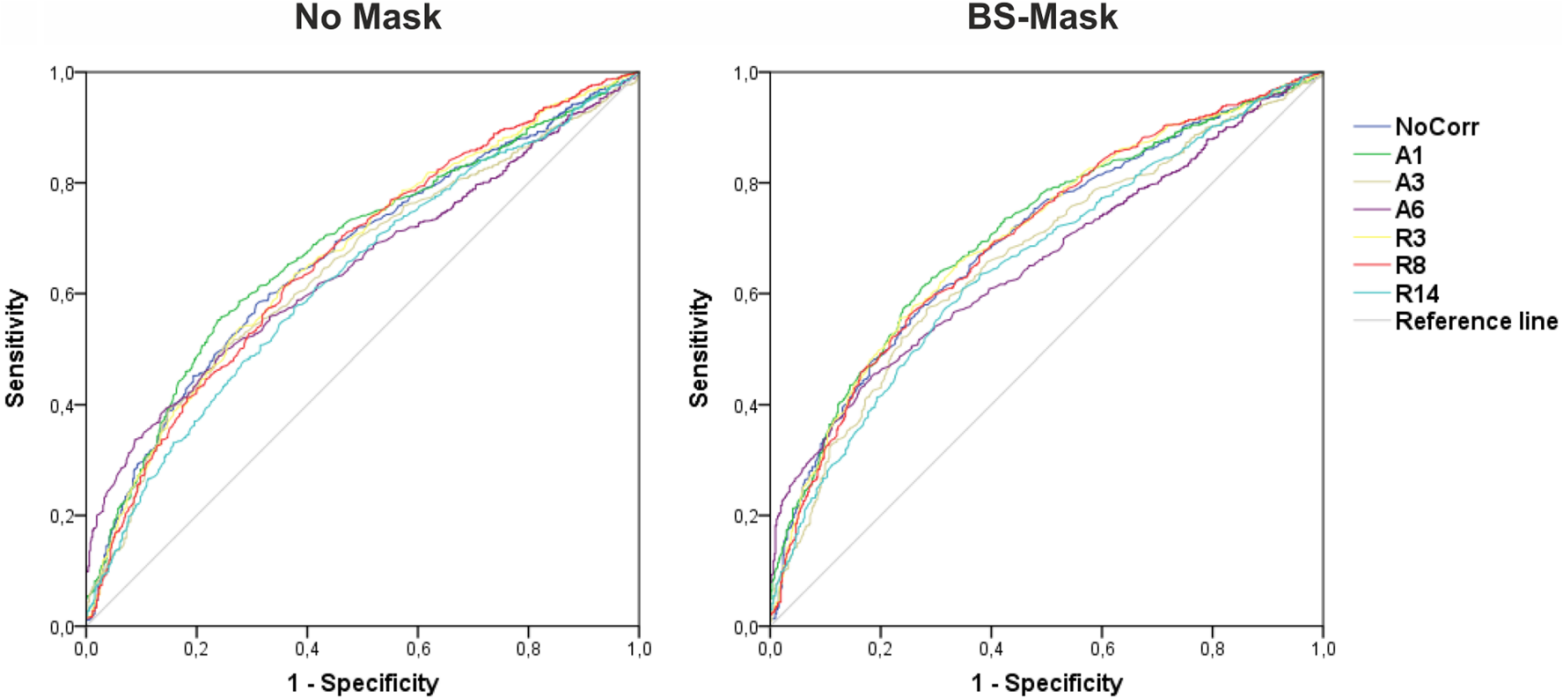
Receiver-operating characteristic (ROC) curves showing true positives (positive *T* values within the target ROI, Sensitivity) and false positive (positive *T* values in the control ROI, 1-Specificity) for all physiological noise correction methods without masking and with masking of the brainstem (BS-Mask). *A*  aCompCor, *R* RETROICOR

The ROC curves showing true positives (positive *T* values within the target ROI, Sensitivity) and false positives (positive *T* values in the control ROI, 1-Specificity) for all physiological noise correction methods are depicted in Fig. 3. The masking procedures resulted in steeper curves, indicating higher sensitivity and specificity compared to no masking (compare No Mask and BS-Mask, Fig. 3). The ROC curves for A3, A6, and R14 lie closest to the 45-degree reference line pointing to a low accuracy of these methods. These observations are supported by the values for the area under the curve (AUC) as a combined measure for sensitivity and specificity (Table 1). For all masking procedures, aCompCor with one regressor (A1, CSF time series) resulted in the highest AUC values. The masking procedures, in particular applying the brainstem mask, increased AUC values in all physiological noise correction methods. Correspondingly, BS-Mask + A1 emerged as the best model, with an AUC value of .709, whereas the base model (no mask, no physiological noise correction) yielded an AUC value of .665. While R3 and R8 had comparable values to no physiological noise correction, applying more regressors (R14) led to lower AUC values. ACompCor with 3 and 6 dimensions resulted in lower AUC values as the base model as well.

**Table 1:** AUC values (95% confidence intervals) depending on masking and correction procedure

	No Corr	A1	A3	A6	R3	R8	R14
No mask	0.665 (0.636–0.693)	0.679 (0.651–0.707)	0.645 (0.617–0.674)	0.646 (0.618–0.674)	0.667 (0.638–0.695)	0.664 (0.636–0.693)	0.628 (0.598–0.657)
CSF mask	0.676 (0.648–0.704)	0.701 (0.674–0.728)	0.642 (0.613–0.671)	0.625 (0.597–0.653)	0.680 (0.652–0.708)	0.671 (0.643–0.700)	0.641 (0.613–0.670)
BS-mask	0.696 (0.669–0.723)	0.709 (0.682–0.736)	0.666 (0.638–0.695)	0.658 (0.630–0.685)	0.704 (0.676–0.731)	0.700 (0.672–0.727)	0.657 (0.629–0 685)

### Reproducibility and reliability

**Fig. 4:**
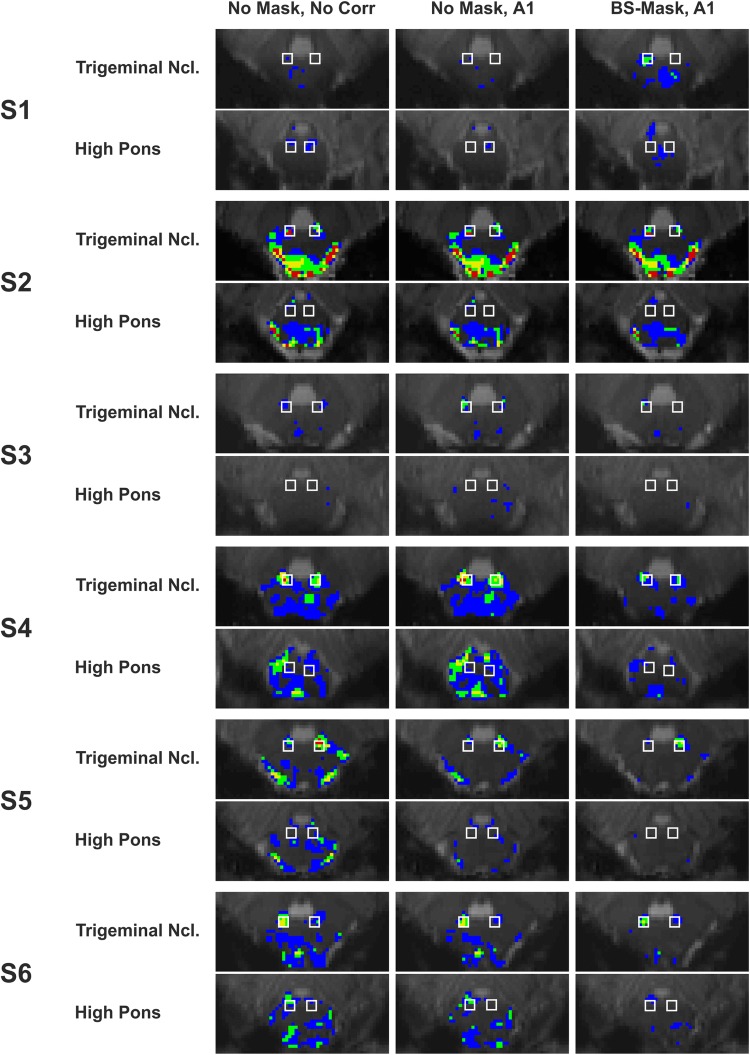
Overlapping activation (FDR 0.05) in four (red), three (yellow), and two sessions (green) and activation found in one session of the four sessions (blue) in slices containing the target ROI (Trigeminal Ncl.) and control ROI (High Pons; ROI boundaries delineated by white squares) for all subjects (S1–S6). The base model (No Mask, No Corr) is compared to aCompCor with one regressor (A1) with and without brainstem masking (BS-Mask). For better comparability, only voxels within the brainstem masks are displayed. Images are shown in neurological convention (left = left)

The analyses of reproducibility and reliability were focused on the masking procedure and the physiological noise correction with the highest improvements regarding the AUC values compared to the base model. Thus, the impact of ACompCor with one regressor (A1) in combination with and without brainstem masking was compared to the base model regarding the Overlap Index (OI) as a measure of reproducibility between sessions in each subject and regarding the ICC for testing reliability over subjects and sessions. In Fig. 4, the activation overlap between the sessions (FDR 0.05 corr.) for each subject is depicted for the base model in comparison with A1 with and without brainstem masking. In the trigeminal target ROI, several voxels showed overlapping activation in four (red), three (yellow), and two (green) sessions, whereas activation in the control ROI was generally low and mostly found in only one session (blue). This inconsistent activation was reduced in most subjects when applying the brainstem mask, particularly in regions adjacent to the CSF. Please note that for a better comparability between the methods, only the voxels within the individual brainstem mask are displayed in Fig. 4.

Regarding the reproducibility, applying aCompCor with one regressor (A1) turned out to be beneficial for all subjects; in three subjects (S1–S3), the highest OI values in the trigeminal ROI (bold numbers in Table 2) were found in combination with the brainstem mask and in the other subjects (S4–S6) in combination with no masking. Two subjects (S1 and S3) showed no activation overlap in the base model (No Mask, No Corr), but with A1 and the brainstem mask the OI values increased noticeably (> 0.667). Correspondingly, the average OI for the trigeminal ROI over all subjects was highest with the brainstem masking in combination with A1 (Table 3).

**Table 2:** Overlap index for all subjects (S1–S6) in the target and control ROI with and without brainstem masking (BS-Mask) combined with no physiological noise correction (No Corr) and ACompCor with one regressor (A1)

	Masking	Correction	S1	S2	S3	S4	S5	S6
Trigeminal Ncl. (Target ROI)	No mask	No Corr	0.000	0.674	0.000	0.900	0.766	0.838
		A1	0.167	0.641	0.600	**0.921**	**0.868**	**0.902**
	BS-mask	No Corr	0.250	0.625	0.000	0.615	0.795	0.803
		A1	**0.765**	**0.719**	**0.667**	0.607	0.781	0.775
High Pons (Control ROI)	No mask	No Corr	0.000	0.500	0.000	0.189	0.000	0.654
		A1	0.000	0.500	0.000	0.375	0.000	0.750
	BS-mask	No Corr	0.333	NaN	0.000	0.000	0.000	0.167
		A1	0.000	NaN	0.000	0.000	NaN	0.000

**Table 3:** Mean overlap index (standard deviation) and ICC values (95% confidence intervals) for the target ROI with and without brainstem masking (BS-mask) combined with no physiological noise correction (No corr) and ACompCor with one regressor (A1)

	Overlap index		ICC values			
			% of activated voxels		Mean *T* values	
	No corr	A1	No corr	A1	No corr	A1
No mask	0.530 (0.381)	0.683 (0.263)	0.543 (0.125–0.901)	0.759 (0.430–0.955)	0.603 (0.189–0.918)	0.769 (0.441–0.958)
BS-mask	0.515 (0.294)	0.719 (0.064)	0.559 (0.143–0.906)	0.675 (0.288–0.937)	0.565 (0.144–0.908)	0.645 (0.254–0.929)

In the control, ROI in the high pons the activation overlap was generally low and only in two subjects were the OI values ≥ 0.500 without masking (S2 and S6), but these decreased when applying the brainstem mask (Table 2). For brainstem masking in combination with A1, all subjects displayed no activation overlap in the control ROI between the sessions (OI = 0.000 or NaN in cases without any significant activation in none of the sessions).

The ICC analysis revealed that the most reliable results for both measures of trigeminal activation (percentage of activated voxels and mean *T* values within the trigeminal ROI) were found when applying aCompCor with one regressor (A1), yielding the highest ICC values (> 0.75) without masking. Applying the brainstem mask did not consistently improve reliability measures (Table 3).

### Task-related motion and physiological traces

**Fig. 5:**
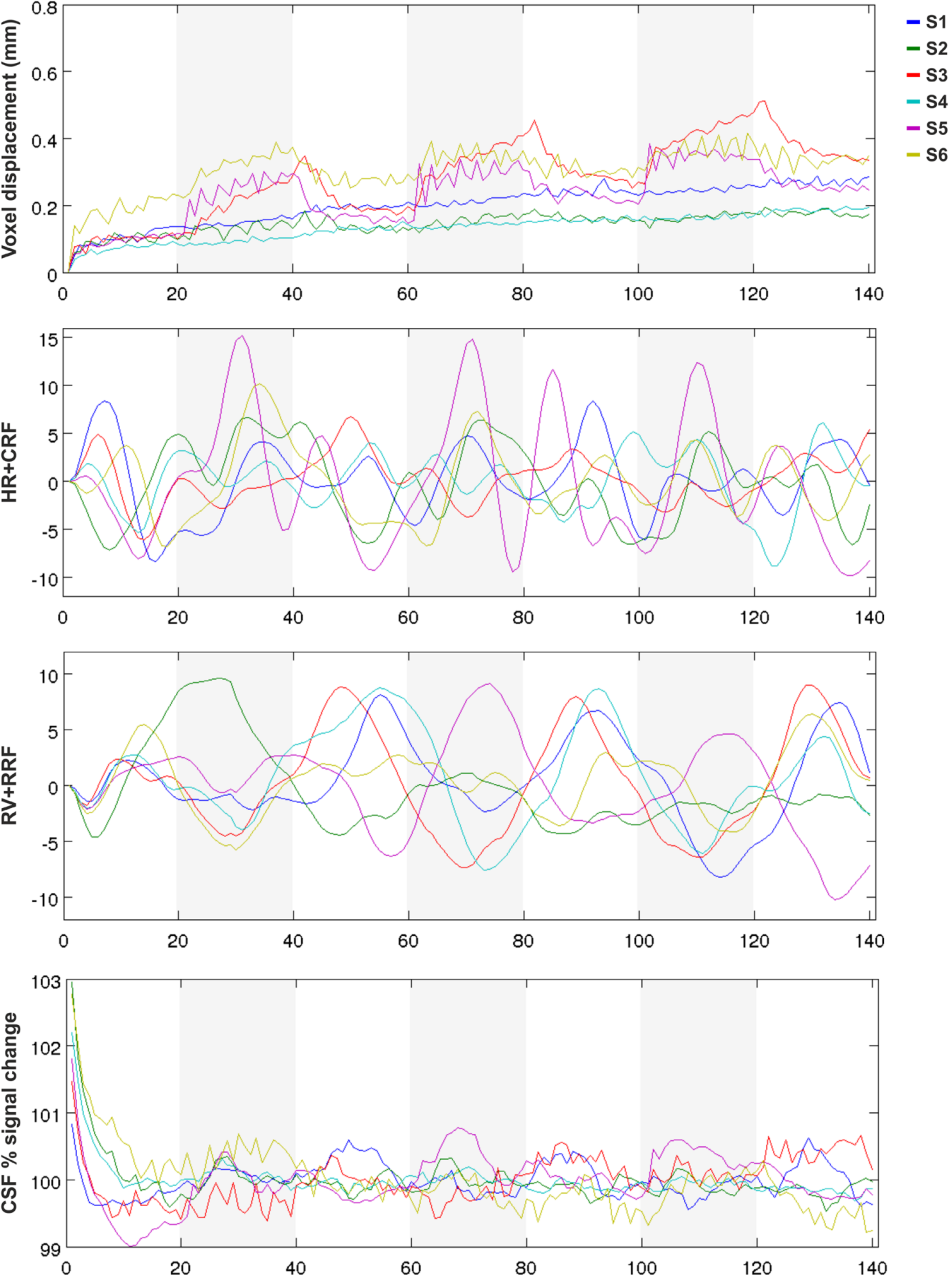
Voxel displacement (root-mean-square of the translation parameters relative to the first volume), heart rate variation convolved with the cardiac response function (HR + CRF), breathing volume variation convolved with the respiration response function (RV + RRF), and percentage of signal change in the cerebrospinal fluid (CSF) plotted against the run duration for each subject (S1–S6, averaged over all runs and sessions). Male subjects (S3, S5, and S6) showed an apparent modulation of the voxel displacement with the motor task (marked in grey)

Calculations of the root-mean-square of the translation parameters revealed that all subjects exhibited a low voxel displacement (VD) averaged over all runs and sessions (< 0.32 mm). However, when plotting the average VD against the run duration, task-related signal modulations were observed in all male subjects (S3, S5, S6). In these subjects, the VD values increased during the movement blocks (20–40 s, 60–80 s, and 100–120 s) about 0.1–0.2 mm on average (Fig. 5). Regressors modelling the effect of heart rate variation (HR + CRF) and respiration volume variation (RV + RRF) on the BOLD response did not show an apparent modulation with the jaw-clenching task. The percentage of signal change in the CSF mask that was used as A1 regressor also revealed no systematic relation with the functional task.

## Discussion

Contrary to the previous functional imaging, studies targeting the human brainstem this work focused on improving functional localization on a single-subject level. By systematically comparing different physiological noise correction methods with respect to activation sensitivity, specificity, reproducibility, and reliability, we provide an optimized acquisition and processing approach to detect brainstem nuclei activation in individual subjects.

### Main findings

The systematic comparison of different physiological noise correction methods showed that the best sensitivity and specificity were achieved for brainstem masking combined with regression of the CSF time series (aCompCor with one regressor, A1). Importantly, this result is not restricted to a certain threshold; the ROC analysis was used to examine sensitivity (active voxels in the target ROI) and specificity (non- active voxels in the control ROI) over all positive *T* values observed in all ROI voxels. The resulting area under the curve (AUC) reflects a combined measure of sensitivity and specificity and—as the ROI definition is based on clear neuroanatomical concepts—may be regarded as an indicator for data validity. With the best model (A1 with the brainstem mask), the AUC value increased to 0.709 compared to the base model (no mask, no physiological noise regressors) with an AUC value of 0.665. The increased specificity of trigeminal activation and a clear reduction of artifacts are apparent when comparing individual activation overlap maps between different models (Fig. 4).

ACompCor with one regressor was also found to increase the reproducibility between sessions and the reliability over subjects and sessions of the trigeminal motor nucleus activation. When applying this correction method, the ICC values increased from 0.543 (base model) to 0.759 for the percentage of activated voxels and similarly for the mean *T* value. The average Overlap Index value (OI) as a measure for reproducibility increased from 0.530 to 0.683 with this method and even more when adding the brainstem masking. That is, including just one additional regressor in the statistical model resulted in a reliability gain of more than 20% and an increase in reproducibility of about 15%. Unlike the results for the AUC and average reproducibility measures, however, a benefit of brainstem masking was not observable for the ICC values. The following sections provide a more detailed assessment of the effects of the different physiological noise correction methods and masking procedures.

### Comparison of the physiological noise correction methods

Including physiological noise, regressors in the statistical model reduced signal variability and generally resulted in a decrease in activation measures (mean *T* value and percentage of activated voxels). For RETROICOR and aCompCor with one regressor (A1), this effect was modest, but aCompCor with more regressors (A3 and A6) led to a marked activation decrease. For the model A6, the loss of sensitivity was dramatic and activation at higher thresholds diminished almost completely. This resulted in the unusual appearance of the ROC curve for A6 which implied that the model was better for low *T* values than for higher ones (that probably reflect “real” activation). The benefit of A1 was evident in all data quality measures assessed here, sensitivity and specificity (AUC), reproducibility (OI), and reliability (ICC). The RETROICOR-based approaches showed some improvements regarding the AUC values compared to no physiological noise correction, but were inferior to the A1 method. Apparently, the time series in the CSF mask captured the most important physiological noise information, probably due to the short TR (1 s). Therefore, using physiological recordings might be useful for fMRI experiments with longer repetition times.

### Impact of the masking procedures on brainstem activation

Applying the brainstem mask led to increased mean *T* values and to higher values for the percentage of activated voxels in the trigeminal ROI, whereas these measures of activation were relatively unaffected by the masking in the control region in the high pons. This implies a specific benefit of the masking on the activation of interest, probably by avoiding the superimposition of CSF signals on the motor nuclei activation. Correspondingly, the AUC values increased when applying masking procedures. Although some benefits of the CSF mask were observable, those effects were rather small compared to brainstem masking. The reason for this might lie in the construction of the CSF mask, which was derived from the automated segmentation of the mean EPI, resulting in a probabilistic assignment of the voxels to tissue classes such as CSF. We set the probability threshold for the CSF mask to 0.25 for all subjects, resulting in a reasonable coverage of the CSF on visual inspection (Fig. 1). However, the delimitation between brainstem tissue and CSF was rather coarse compared to the manual definition of the brainstem which apparently led to a smearing of parts of the CSF signal into the activation of interest.

Given the increased sensitivity and specificity due to the brainstem masking, one would expect a benefit for the reproducibility and reliability as well. For the trigeminal ROI, the brainstem masking resulted in higher OI values in three out of six subjects, while in the other subjects, the masking was not beneficial for the reproducibility. Apparently, the brainstem masking led to an increased variability between the subjects that is mirrored by the decreased ICC values as a measure for the inter- and intra-subject agreement of trigeminal activation. In contrast, for the control ROI, the OI results reveal a consistent decrease in false positive activation overlap indicating a higher specificity, similar to the AUC analysis, when applying the brainstem masking.

### Limitations

#### ROI selection

The volume of the target ROI was larger than the expected volume of the trigeminal nucleus to account for individual variation in the real location of the nucleus. This is mirrored by the fact that fewer than 40% of voxels showed activation in the target ROI (FDR 0.05 corr.). Despite this relatively large target ROI, trigeminal activation lay partly outside the ROI boundaries in some subjects (see Fig. 4), underlining the inter-individual variability of brainstem anatomy and vascularization. However, trigeminal voxel clusters were clearly separated from noise and were well reproducible. The selection of the control ROI is crucial for calculating the specificity of the different methods. For the correct estimation of the AUC, the control ROI must have the same size as the target ROI. It is also required to not be co-activated during the jaw-clenching task, as an estimation of non-functional physiological noise in the brainstem is desirable. The brainstem contains several motor and sensory nuclei that make the definition of such an area challenging and led to our choice to select an area in the high pons. The control ROI lies in close vicinity of the locus coeruleus yielding a risk of capturing signals related to attention modulation (Schwarz and Luo 2015). However, we found very low activation and hardly any activation overlap between sessions in the high pons ROI. These results indicate no systematic recruitment of this area during the motor task and support its suitability as a control ROI.

#### Functional image quality

Ultra-high field increases signal-to-noise ratio, but increases other artifacts such as distortions which can only partially be corrected using “static” distortion correction (Jezzard and Balaban 1995; Robinson and Jovicich 2011). “Dynamic” distortion correction, in which a time series of B_0_ field maps are calculated from the phase images of each volume in the fMRI time series, has been shown to be robust to motion and breathing-related changes in the field (Dymerska et al. 2018), and could offer improved BOLD sensitivity in the brainstem. Coregistering the functional images to an anatomical image was beyond the scope of this work, but might be expected, in the light of the previous findings, to be impeded by these distortions (Cusack et al. 2003; Gartus et al. 2007). In addition, in two subjects (Subject 1 and 4), anterior parts of the pons were affected by signal dropouts—probably due to dental fillings. Despite the fact that target and control ROI were not affected by these signal dropouts (as evidenced by tSNR values which were comparable in both ROIs), this might be detrimental for other research targets in the brainstem. The images in this study were acquired with a head coil which yields relatively low signal in caudal regions due both to poor RF transmit (B1^+^) efficiency and limited coverage by receive coils. The use of head–neck coils and parallel transmission would probably result in a higher signal-to-noise ratio and provide an improved sensitivity for brainstem nuclei activation.

#### Motion and physiological confounds

Voxel displacement due to subject motion during the runs was quite low on average, but showed an apparent modulation with the motor task in the three male subjects. Although the amplitudes of these modulations during the movement blocks were low (ranging between 0.1 and 0.2 mm) using the motion parameters as nuisance regressors might have reduced the task-related BOLD signal in these subjects. On the other hand, some suspected motion artifacts at the anterior border of the pons found in the subjects S2, S5, and S6 endured despite this motion correction. In the male subjects S5 and S6, for whom task-related motion was evident in the voxel displacement curves, applying the brainstem mask and A1 clearly reduced these artifacts. In subject 2, neither the motion nor the physiological traces revealed an obvious modulation with the task. However, a more complex relation between jaw clenching and systemic, vascular, or respiratory functions is conceivable. In general, the effect of pulse and respiration on the BOLD response as modelled by the HR + CRF and RV + RRF functions as well as the percentage of signal change in the CSF mask (A1 regressor) did not show consistent relations with the jaw-clenching task.

### The potential of clinical brainstem fMRI

So far, functional imaging studies reporting aberrant brainstem activation in clinical populations have been group studies and none have used acquisition or processing strategies that were optimized for the brainstem. For example, Hacker et al. (2012) used a whole-brain resting-state approach in patients with Parkinson’s disease (PD) and found a lower striatal correlation with brainstem areas in patients compared to healthy controls. Holiga et al. (2015) demonstrated that changes in functional connectivity in the brainstem were associated with motor improvement due to the implantation of deep brain stimulation electrodes. Altered functional connectivity of brainstem areas was also shown in chronic pain. In patients with cluster headache, a significantly higher activation in several brainstem regions was found during the pain attack compared to a pain-free state (Morelli et al. 2013). In migraineurs, increased sensitivity to pain-related stimuli has been found to be related to altered functional connectivity of the periaqueductal gray and other subcortical and cortical pain processing areas (Schwedt et al. 2014; Marciszewski et al. 2018). These studies demonstrate the clinical significance of functional imaging of the brainstem, in particular in movement disorders and chronic pain. However, these studies were whole-brain approaches with relatively low spatial resolution (voxelsize > 9 mm^3^) and large smoothing kernels, suboptimal for the investigation of small brainstem nuclei. This insufficient spatial resolution hinders a proper localization of brainstem activation (Holiga et al. 2015) and increases the partial volume effect. The resulting decrease in sensitivity for small-activated regions impedes the search for brainstem nuclei activation in general and, to an even greater extent, differences between experimental groups. The present findings and previous studies (Beissner et al. 2014; Harvey et al. 2008; Hutton et al. 2011) have demonstrated the impact of physiological noise on brainstem activation measures. The potential of physiological fluctuations to confound the BOLD signal should thus be controlled for in future clinical applications of brainstem fMRI.

With pathological tissue, morphological and functional changes often limit the efficacy of technologies which have been developed for standard group fMRI studies. For clinical applications, it is of utmost importance to achieve not only sensitive but also reliable brainstem signals on an individual level (Beisteiner 2017). The feasibility of localizing brainstem nuclei on a single-subject level offers promising possibilities to apply brainstem fMRI in clinical settings. A better characterization of functional aberrations in the brainstem would further our pathophysiological understanding of all diseases affecting the brainstem, particularly movement disorders. Braak et al. (2003) observed that PD pathology follows distinctive spatio-temporal patterns, with the first two stages affecting the medulla oblongata followed by a spreading along the pons and midbrain associated with the occurrence of the first motor signs. The brainstem is thus regarded as a promising target region to find preclinical markers for PD and to test therapeutic effects of potential neuroprotective agents or brain stimulation techniques (Holiga et al. 2015; Tison and Meissner 2014). Moreover, a differential involvement of brainstem neuropathology has been observed in atypical parkinsonian disorders such as corticobasal degeneration, multiple system atrophy, and progressive supranuclear palsy (Jellinger 2008; Levin et al. 2016). Given that the clinical definition of these syndromes is difficult (Levin et al. 2016; Stamelou and Hoeglinger 2013), brainstem fMRI might have potential as a future in vivo tool for differential diagnosis in movement disorders.

## Conclusion

Brainstem motor nuclei activation can be reliably identified using high-field fMRI with optimized acquisition and processing strategies—even on single-subject level. Applying specific physiological noise correction methods improves reproducibility and reliability of brainstem activation encouraging future clinical applications.
